# Peripheral vascular dysfunction and the aging brain

**DOI:** 10.18632/aging.205877

**Published:** 2024-05-22

**Authors:** Devin Wahl, Zachary S. Clayton

**Affiliations:** 1 Department of Health and Exercise Science and Center for Healthy Aging, Colorado State University, Fort Collins, CO 80523, USA; 2 University of Colorado Anschutz Medical Campus, Department of Medicine, Division of Geriatric Medicine, Aurora, CO 80045, USA

**Keywords:** peripheral vascular health, cerebrovascular health, cognitive function, dementia

## Abstract

Aging is the greatest non-modifiable risk factor for most diseases, including cardiovascular diseases (CVD), which remain the leading cause of mortality worldwide. Robust evidence indicates that CVD are a strong determinant for reduced brain health and all-cause dementia with advancing age. CVD are also closely linked with peripheral and cerebral vascular dysfunction, common contributors to the development and progression of all types of dementia, that are largely driven by excessive levels of oxidative stress (e.g., reactive oxygen species [ROS]). Emerging evidence suggests that several fundamental aging mechanisms (e.g., “hallmarks” of aging), including chronic low-grade inflammation, mitochondrial dysfunction, cellular senescence and deregulated nutrient sensing contribute to excessive ROS production and are common to both peripheral and cerebral vascular dysfunction. Therefore, targeting these mechanisms to reduce ROS-related oxidative stress and improve peripheral and/or cerebral vascular function may be a promising strategy to reduce dementia risk with aging. Investigating how certain lifestyle strategies (e.g., aerobic exercise and diet modulation) and/or select pharmacological agents (natural and synthetic) intersect with aging “hallmarks” to promote peripheral and/or cerebral vascular health represent a viable option for reducing dementia risk with aging. Therefore, the primary purpose of this review is to explore mechanistic links among peripheral vascular dysfunction, cerebral vascular dysfunction, and reduced brain health with aging. Such insight and assessments of non-invasive measures of peripheral and cerebral vascular health with aging might provide a new approach for assessing dementia risk in older adults.

## INTRODUCTION

Advancing age is the largest non-modifiable risk factor for most chronic diseases including cardiovascular disease (CVD) and dementia [1]. Many of these diseases arise in parallel and are thus termed ‘co-morbidities of aging’ [2, 3]. In particular, growing evidence suggests that CVD and dementia are correlatively linked in that CVD risk in mid-life is associated with memory decline and subsequent dementia diagnosis in older age (e.g., >70 years of age) [4, 5]. In parallel, peripheral vascular dysfunction (e.g., large elastic artery [aorta and carotid arteries] stiffening and endothelial dysfunction) is a main contributor to the development of CVD with aging [6, 7], and growing evidence suggests that peripheral vascular dysfunction may also be associated with cognitive decline and mild cognitive impairment (MCI), which increases the risk for dementia [8]. As such, assessment of peripheral vascular health/function may be a viable and non-invasive method to predict cognitive outcomes and assess dementia risk.

Interestingly, the associations between common non-invasive measures of CV health (e.g., blood pressure) and risk of dementia are not well-established. For example, studies have demonstrated that lower systolic blood pressure is associated with reduced dementia risk in older adults (60-70 years of age), perhaps by protecting white matter integrity [9]. On the contrary, other studies have suggested that higher blood pressure might be beneficial to protect against cognitive decline and subsequent dementia diagnosis, presumably by increasing blood flow to the brain and preventing hypoperfusion [10]. Therefore, it is an opportune time to consider whether alternative and less commonly used measures of peripheral vascular health, which are closely associated with age-related CVD and described in detail below, might be used to predict cognitive dysfunction and dementia risk more accurately.

## Associations between Dementia and Cerebrovascular Dysfunction

**Figure 1:**
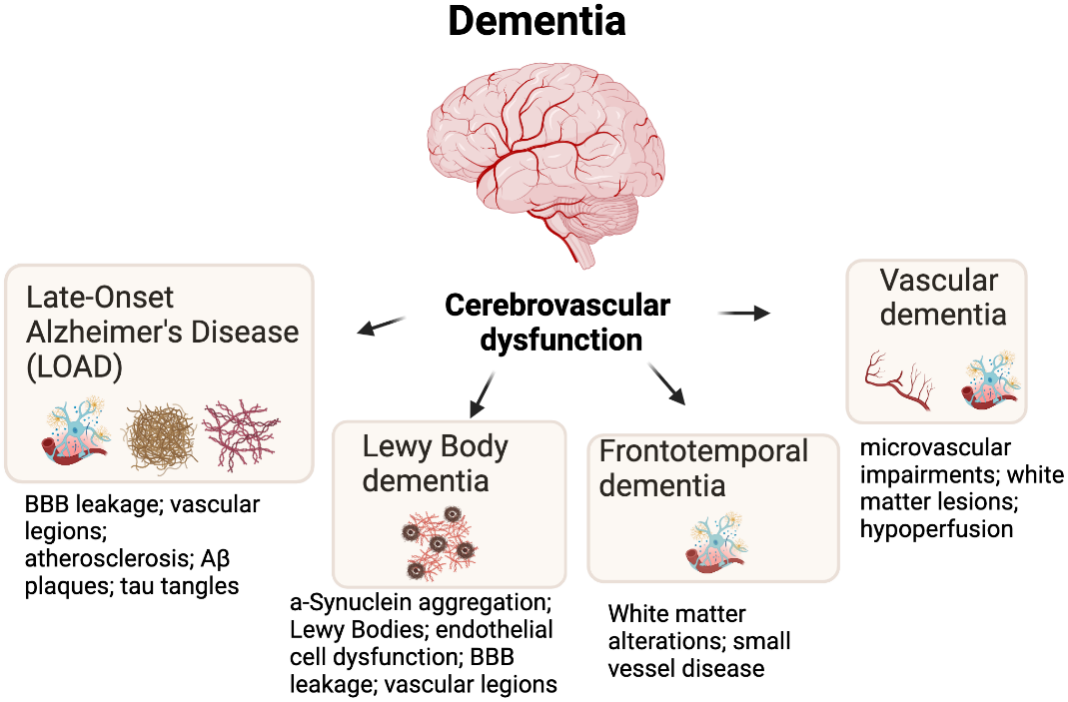
**Cerebrovascular dysfunction is an underlying feature of most major types of dementia.** Additional and key hallmarks are shown for each dementia subtype. Abbreviations: BBB=blood brain barrier; Aβ=Amyloid beta.

Dementia is commonly defined as a condition that is progressive and affects various domains of cognitive function. Advanced dementia impairs function (i.e., the ability to perform common day-to-day activities) and greatly reduces memory, thinking, learning, comprehension, and decision making [11]. There are many types of dementia with various genetic, cellular, and pathological underpinnings, and the purpose of this review is not to describe these types of dementia in detail. However, vascular dysfunction in the brain consistently emerges as a common feature among major types of dementia [12, 13]. Below, we very briefly describe the most common subtypes of dementia and how cerebrovascular dysfunction (i.e., dysfunction of the vasculature of the brain) relates to each type (Figure 1). We also describe how ROS-related oxidative stress is associated with these types of dementia.

### Late-onset Alzheimer’s disease

The most common type of dementia is Late-Onset Alzheimer’s Disease (LOAD) which accounts for ~50-60% of all dementia cases [14]. Neuropathologies that are associated with Alzheimer’s disease (e.g., Aβ plaques and tau neurofibrillary tangles) are closely linked to cerebrovascular dysfunction by inducing vascular lesions, blood-brain barrier (BBB) disruption, and atherosclerosis [15]. Pathological Aβ and hyperphosphorylated tau are also linked to excessive oxidative stress, which may further drive the cycle of neuronal dysfunction and cognitive impairment [16, 17].

### Lewy body dementia

Lewy body dementia accounts for ~3-7% of all dementia cases and is closely associated with the pathological aggregation of the α-Synuclein (Lewy bodies) and to a lesser extent Aβ plaques [18]. The role of pathogenic α-Synuclein in cerebrovascular dysfunction is complex and largely misunderstood, but recent evidence points towards the fact that α-Synuclein misfolding and aggregation triggers neuroinflammatory responses, ROS production, and mitochondrial dysfunction [19]. In fact, some studies on postmortem tissue indicate that cerebrovascular lesions are even associated with Lewy body aggregation [20], but future studies are needed to determine more conclusive links between cerebrovascular dysfunction (and related mechanisms) and α-Synuclein aggregation.

### Frontotemporal dementia

Frontotemporal dementia (FTD) accounts for ~3-26% of all dementias and results in progressive neuron loss in frontal and temporal lobes. This neuron loss and pruning (e.g., loss of synapses) is associated with behavioral abnormalities (e.g., apathy, agitation, and decline in socially acceptable behavior) [21]. The links between FTD and cerebrovascular dysfunction remain understudied, but some cases of FTD are strongly associated with white matter alterations, cerebrovascular dysfunction, and small-vessel (e.g., coronary arteries) disease in the brain [22]. As with most types of dementia, FTD is also closely associated with excessive ROS-related oxidative stress [23].

### Vascular dementia

Vascular dementia, a form of dementia caused by diseased intracranial arteries (e.g., infarction; chronic hypertension), accounts for ~4-6% of all dementias [24, 25]. Evidence suggests that cerebral hypoperfusion may be main another cause of vascular dementia, which can lead to hypoxia and blood-brain barrier breakdown [13]. Furthermore, vascular dementia is associated subcortical white matter lesions, microvascular impairments in the brain, and disrupted neuronal network activity [24]. As with the other types of dementia, vascular dementia is closely associated with ROS-related oxidative stress [26].

## Links between Cerebral Vascular and Peripheral Vascular Dysfunction

Treatments (described below in more detail) aimed at reducing peripheral vascular risk factors, such as aerobic exercise and certain dietary patterns, have been shown to attenuate dementia incidence and progression [13]. These mechanisms largely involve lower ROS-related oxidative stress and inflammation, improved mitochondrial function, and reduced burden of cellular senescence. Emerging evidence suggests that these mechanisms also contribute to cerebrovascular dysfunction, suggesting that measurements of peripheral vessel function are good indicators of brain health/function [27].

### Cerebrovascular dysfunction

The capacity of the cerebrovasculature to coordinate blood flow to tissue demand and remove toxic compounds is essential for maintaining brain homeostasis and cognitive health [28, 29]. A critical function of cerebral vessels is to dilate and constrict in response to changes in blood flow demand, and as such, reductions in cerebral blood flow may reflect dysfunction of the cerebrovasculature [28, 29]. There is clear cerebrovascular dysfunction with aging [30]; however, the exact mechanisms mediating this dysfunction are incompletely understood. Emerging evidence suggests that cerebrovascular dysfunction with aging could be due to the way in which blood flow is delivered to the brain [31, 32]. For example, with advancing age, there is increased cerebrovascular pulsatility, which describes the variation of blood flow to the brain within each cardiac cycle [33]. Higher cerebral pulsatility can damage the cerebrovasculature and structures within the brain, ultimately leading to cerebrovascular diseases and reduced cognitive function [33]. As such, mitigating the increase in cerebral pulsatility with advancing age or in a state of advanced age, holds promise for preserving and/or improving brain health and potentially reducing risk of dementia.

### Peripheral vascular dysfunction

Peripheral vascular dysfunction, as defined above (e.g., large elastic artery stiffening and endothelial dysfunction), occurs with advancing age [7]. The large elastic arteries expand and recoil with each bolus of blood ejected from the left ventricle during systole. The process of expanding and recoiling allow for dampening of the oscillatory pulse of blood that is ejected into the arterial system and aids in the outward flow of blood into the peripheral circulation and helps maintain perfusion of the heart during diastole [34]. The pulsatility-dampening effect of large elastic arteries is critical for reducing the transmission of harmful high pulsatile pressures to low-impedance, high flow sensitive organs, such as the brain [34].

#### Large elastic artery stiffness

Age-related large elastic artery stiffening occurs mainly as a result of degradation of the load bearing arterial wall protein, elastin, which is primarily responsible for the expansion and recoiling of the large elastic arteries during systole [34]. Elastin degradation consequently results in deposition of collagen in the arterial wall (i.e., fibrosis), ultimately creating a stiffer extracellular matrix [34]. The most well-established cellular/molecular mechanisms mediating adverse arterial wall remodeling is excessive production of ROS (relative to endogenous antioxidant defenses) and chronic low-grade inflammation [35].

Large elastic artery stiffness can be assessed *in vivo* as aortic pulse wave velocity (PWV), which is an assessment of the regional speed of the pulse wave generated by the heart when blood is ejected into the arterial system [36]. Aortic PWV, measured as carotid artery to femoral artery (carotid-femoral) PWV is the reference standard non-invasive *in vivo* assessment of aortic stiffness in humans [36]. In rodents, aortic PWV is measured as the PWV between the aortic arch and the abdominal aorta [35]. The local distensibility of the carotid artery can also be determined in humans by assessing carotid artery compliance (change in artery diameter for a given change in arterial pressure) [37].

#### Vascular endothelial function

The vascular endothelium is a single layer of cells lining the lumen of blood vessels. The endothelium plays a critical role in the regulation of vascular tone and systemic blood flow, metabolism, thrombosis, immune system function and a variety of other processes, in part via the production of the vasodilatory and mostly vasoprotective molecule nitric oxide (NO) [38]. Mechanical (i.e., blood flow-induced shear stress) and chemical (e.g., acetylcholine [ACh]) stimuli elicit NO production in endothelial cells. Endothelium-derived NO subsequently diffuses to vascular smooth muscle cells, where it activates an intracellular signaling cascade leading to vascular smooth muscle relaxation and vasodilation (endothelium-dependent dilation [EDD]) [38]. Like mechanisms mediating age-related large elastic artery stiffening, the underpinnings of reduced NO bioavailability and endothelial dysfunction with aging are primarily explained by excessive ROS-related oxidative stress and inflammation [35]. Moreover, NO-mediated endothelial dysfunction can promote large elastic artery stiffening by inducing a relative state of vasoconstriction in vascular smooth muscle cells [35]. In addition to reducing NO bioavailability, excessive ROS production and inflammation can increase the expression of endothelin-1 (ET-1) [39]. The increased expression and production of ET-1 can further reduce the bioavailability of NO and promote adverse arterial wall remodeling [39].

**Figure 2:**
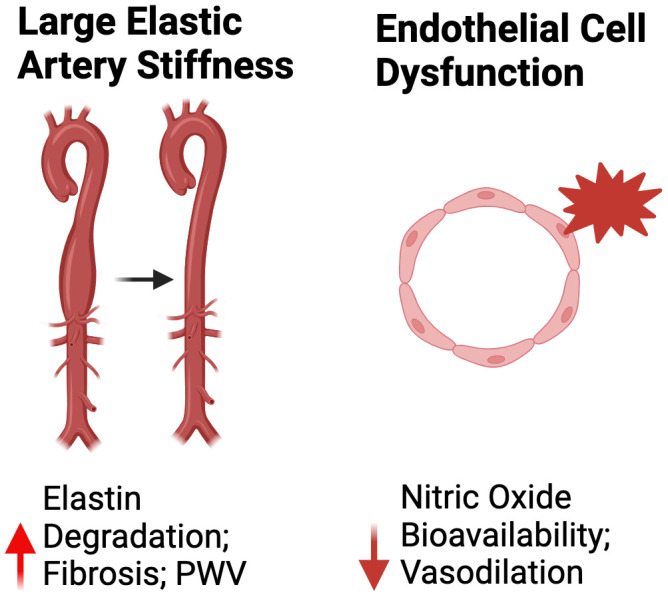
**Mechanisms of peripheral vascular dysfunction.** Abbreviations: PWV, pulse wave velocity.

The gold-standard non-invasive *in vivo* assessment of NO-mediated vascular endothelial function in humans is brachial artery flow-mediated dilation (FMD_ba_), which consists of determining the change in brachial artery diameter in response to a blood flow stimulus (which results in a shear rate-induced release of NO) [38]. NO-mediated EDD can also be assessed in humans as the change in blood flow in response to intra-arterial infusion of acetylcholine (ACh) or in isolated artery segments of rodents following exposure to ACh [40]. In preclinical animal models, endothelial function is commonly assessed by exposing isolated artery segments (e.g., carotid arteries) to ACh [40, 41] (Figure 2).

## Relation between Peripheral Vascular Function and Brain Health

As previously stated, large elastic artery stiffening can increase the pulsatility of blood flow to the brain. Considering the brain is a high-flow organ with low resistance vascular beds, the increase in pulsatility can damage cerebral microvessels and directly promote cerebrovascular dysfunction leading to a state of hypoperfusion of oxygen to the brain and ultimately cognitive impairment [42]. Indeed, a myriad of large cohort studies (n = 205-3207) have consistently demonstrated that large elastic artery stiffening, as measured by aortic PWV, is inversely associated with cognitive function [32, 43–45]. In addition to aortic PWV, aortic stiffness as assessed by carotid artery stiffening, is inversely related to cognitive function in ML/O adults [46–49]. Importantly, large elastic artery stiffening has emerged as an independent predictor of future cognitive impairment in ML/O adults [50].

**Figure 3:**
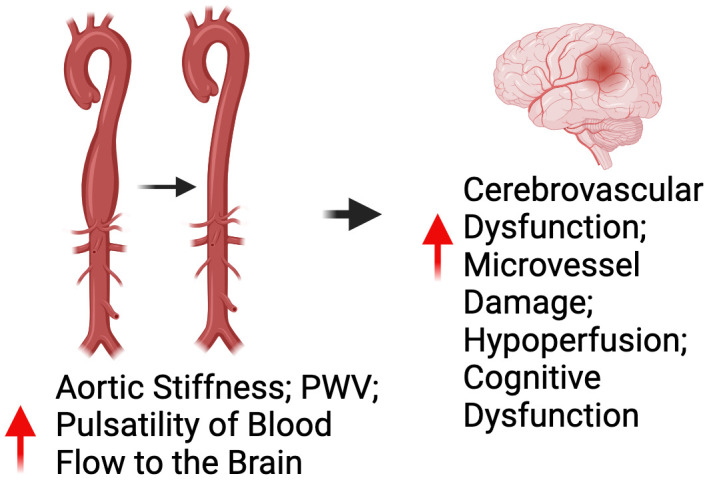
**Relationship between peripheral vascular dysfunction and brain health.** Abbreviations: PWV=pulse wave velocity.

Like large elastic artery stiffening, peripheral vascular endothelial dysfunction, as assessed by flow-mediated dilation, is a measure of peripheral vascular function that is associated with cognitive impairment [51, 52]. However, it is currently unclear whether endothelial dysfunction is an independent predictor of future cognitive impairment. Nevertheless, given the influence of endothelial dysfunction in the progression of large elastic artery stiffening, it is apparent that peripheral vascular dysfunction contributes to cognitive impairment with aging (Figure 3).

## Shared Molecular/Cellular Mechanisms

Although the physiological relation between peripheral vascular dysfunction and brain aging has been well documented, the shared cellular/molecular mechanisms mediating both processes have not been thoroughly reviewed concomitantly. As described throughout this review, excessive production of ROS-related oxidative stress underlies both peripheral vascular dysfunction and brain aging.

**Figure 4:**
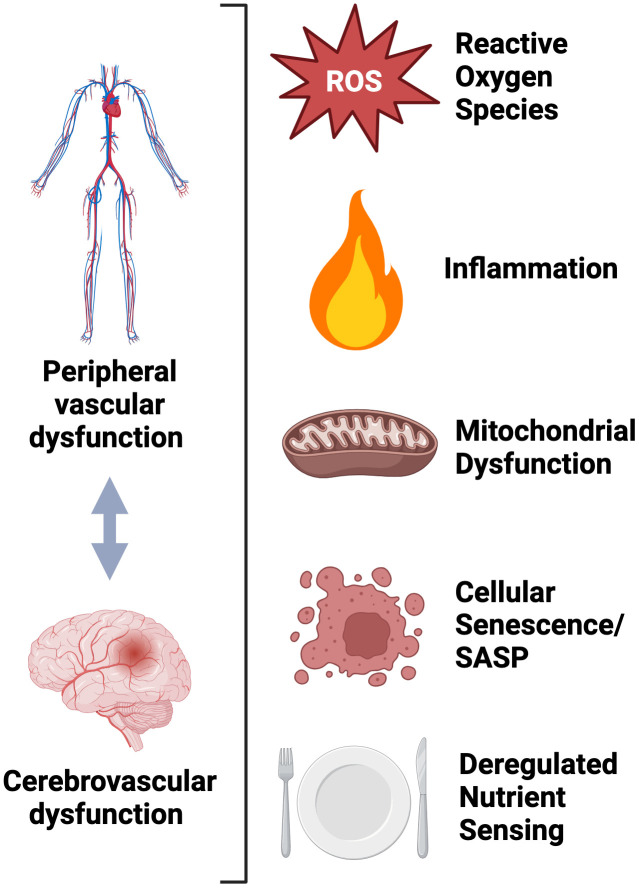
Molecular and cellular mechanisms shared between peripheral and cerebrovascular dysfunction.

Although excessive oxidative stress is a well-established macro-mechanistic process underpinning both peripheral vascular dysfunction and brain aging, the integrative cellular and molecular processes mediating this response are incompletely understood. Herein, we will focus on select “hallmarks” of aging [1] that have been implicated in both peripheral vascular and cognitive dysfunction and are key mediators of excessive ROS production. Moreover, we will discuss these “hallmarks” as putative targets, which could be “aimed at” to improve peripheral vascular and cognitive function with aging. Below, we will focus key aging “hallmarks”: inflammation, mitochondrial dysfunction, cellular senescence and deregulated nutrient sensing (Figure 4).

### Inflammation

Chronic low-grade inflammation, characterized in part as heightened nuclear factor κ B (NF κB) signaling, is a key feature of aging and occurs due to an imbalance between the production of pro- and anti-inflammatory factors [1], which can directly contribute to ROS production [53] and peripheral vascular dysfunction [54]. In old mice, inhibition of NF κB signaling with salisylate (an NF κB inhibitor), has shown to restore carotid artery endothelial function back to young levels. Direct inhibition of NF κB signaling (with salsalate – human corollary of salislyate) lowers aortic stiffness in sedentary normal weight ML/O adults and improves endothelial function in overweight/obese ML/O adults [55, 56]. Furthermore, oral supplementation with the well-established anti-inflammatory compound curcumin has been shown to restore (back to young levels) aortic stiffness and carotid artery endothelial function in old mice [57] and improve endothelial function in ML/O adults (no influence on aortic stiffness) [58]. Together, the results of these studies demonstrate a clear role for chronic low-grade inflammation in mediating peripheral vascular dysfunction with aging.

In addition to the clear effect of inflammation on age-related peripheral vascular dysfunction, inflammation is highly implicated in brain aging [59]. Levels of inflammation in the brain are vastly regulated under normal physiological conditions; however, in states of excessive inflammation (e.g., aging), the brain becomes progressively vulnerable. Excessive inflammation with aging may adversely influence brain function in potentially three different ways: 1) directly via impairment of the blood brain barrier (BBB); 2) indirectly via elevated levels of pro-inflammatory factors in the circulation that can cross the BBB; and/or 3) astrocyte and microglia activation [60]. The BBB is comprised of tight junctions, pericytes, basal membranes and perivascular astrocytes and is a direct conduit between the peripheral circulation and the tissues of the brain. Excessive inflammation can impair BBB integrity by acting on any or all components of the BBB. Moreover, with impaired BBB function, systemic inflammation is more susceptible to crossing into brain tissues, and astrocytes and microglia have greater likelihood of being activated, ultimately causing a state of neuroinflammation. Indeed, with clinical conditions characterized by heightened immune responses (e.g., rheumatoid arthritis and acute infection), or a heightened inflammatory state [61–63], there is elevated risk for dementia. Moreover, dementia risk is higher in patients with genetic conditions that are characterized in part by excessive inflammation (e.g., amyotrophic lateral sclerosis) [64].

### Mitochondrial dysfunction

The signaling functions of vascular mitochondria are thought to be mediated largely by the production of ROS at low, physiological levels [65]. However, dysregulation of mitochondria-derived ROS production also has the potential to lead to pathophysiological sequelae that disrupt other mitochondrial-specific functions, cellular homeostasis, and ultimately peripheral vascular function [65]. Indeed, with aging there is a marked increase in the production of mitochondrial ROS in the vasculature [66, 67] and this excessive production of mitochondria-derived ROS directly contributes to peripheral vascular dysfunction with aging, which has been previously reviewed in detail [65]. Moreover, suppressing tonic excess production of mitochondrial ROS in the vasculature with a mitochondria-targeted antioxidant (e.g., oral MitoQ supplementation) has shown to reverse carotid artery endothelial dysfunction [68, 69] and aortic stiffness (Gioscia-Ryan et al., 2018) in old mice and increase endothelial function and lower aortic stiffness in a pilot clinical trial in ML/O adults [70]. The findings of this pilot trail are currently being translated in a properly-powered placebo-controlled randomized clinical trial (NCT02597023) [71].

Mitochondrial dysfunction, characterized in part by excessive mitochondrial ROS production, is also highly implicated in brain aging [72, 73], which has been reviewed in detail elsewhere [74]. Briefly, the accumulation of mitochondrial ROS has been shown to directly damage neurons in various brain regions, ultimately perpetuating the pathophysiology that leads to the development of Alzheimer’s disease and related dementias [75]. Like peripheral vascular function, MitoQ supplementation promotes healthy brain aging. For example, supplementation with MitoQ inhibits phenotypes of brain aging in mouse [76, 77] and *C. elegans* [78] models of Alzheimer’s disease. Moreover, SS-31 (a mitochondria-targeted antioxidant peptide) has shown to improve cognitive function in old mice [79]. There is an ongoing placebo-controlled randomized clinical trial seeking to determine the efficacy of MitoQ supplementation for improving cognitive function in older adults (NCT06027554).

### Cellular senescence

Cellular senescence is a multi-faceted stress response in which cells undergo a largely permanent cell cycle arrest but remain metabolically active [80]. Key characteristics of senescent cells are an upregulation of cell cycle arrest genes/proteins (e.g., cyclin dependent kinases [p16/p21]) and secretion of pro-inflammatory factors, broadly referred to as the senescence-associated secretory phenotype (SASP) [80]. Physiological levels of cellular senescence aid in processes such as wound healing [81] and cancer suppression [82]; however, with aging there is an excessive accumulation of senescent cells, and this accumulation is thought to promote diseases of aging in part via the SASP [83]. Indeed, cellular senescence and the SASP have shown to directly increase ROS production [80].

An elevated abundance of senescent cells in the vasculature has shown to be inversely related to peripheral endothelial function in ML/O adults [84], and genetic-based clearance of excess senescent cells in old mice has been demonstrated to reverse carotid artery endothelial dysfunction and aortic stiffness [85], ultimately establishing cellular senescence as a viable therapeutic target for improving peripheral vascular function with aging. We and others’ [85, 86] have shown that targeting cellular senescence with synthetic pharmacological-based senolytic therapy (e.g., administration of compounds that can selectively clear senescent cells) can improve peripheral vascular function in old mice, thus, providing essential proof-of-principle efficacy for the potential use of senolytic therapy to improve peripheral vascular function with aging.

Cellular senescence is also implicated in brain aging, which was recently reviewed in detail [87]. For example, select biomarkers of cellular senescence in peripheral blood cells are associated with mild cognitive impairment in older adults [88], and cellular senescence has been shown to directly mediate cognitive function in old rats [89] and mice [90]. Moreover, senescent cells have been demonstrated to accumulate in aged human brain organoids [91] and in brains of mice with accelerated tau burden and the excess accumulation of senescent cells in both models can be suppressed with synthetic pharmacological senolytic therapy. Moreover, senolytic therapy reduces neurofibrillary tangles in mice with accelerated tau burden [92]. Together, these results, like peripheral vascular function, establish cellular senescence as a putative therapeutic target for enhancing brain health with aging.

### Deregulated nutrient sensing

Deregulated nutrient sensing is characterized largely by a reduction in the bioavailability of nicotinamide adenine dinucleotide (NAD^+^) but also consists of reduced abundance and activity of sirtuin enzymes, adenosine monophosphate kinase, and heightened activation of the mammalian target of rapamycin. Of these, reduced NAD^+^ bioavailability has emerged as a highly compelling nutrient sensing-related therapeutic target for improving peripheral vascular and cognitive function with aging, as reduced bioavailability of NAD^+^ is a common manifestation of advancing age and impaired NAD^+^ bioavailability has been linked to peripheral vascular dysfunction and cognitive impairment with aging. Moreover, oral consumption of the NAD^+^ boosting compound nicotinamide riboside (NR) has shown to be well-tolerated, safe and efficacious for increasing NAD^+^ bioavailability in ML/O adults [93]. Another commonly used NAD^+^ boosting compound that has shown to be safe for human consumption is nicotinamide mononucleotide (NMN) [94]. Oral NMN supplementation has been demonstrated to fully reverse carotid artery endothelial dysfunction and aortic stiffening in old mice [95] and in a pilot clinical trial, supplementation with NR has shown to lower aortic stiffness (no influence on endothelial function) in ML/O adults [93]. The findings of this pilot trail are currently being translated in a properly-powered placebo-controlled randomized clinical trial (NCT03821623) [96].

In addition to improving peripheral vascular function with aging, NMN supplementation has shown to attenuate cognitive impairment in old mice [97] and in a rat model of Alzheimer’s Disease [98]. Moreover, supplementation with NR has been demonstrated to restore cognitive function in old mice [99] and in a mouse model of Alzheimer’s disease [100]. However, results supporting the benefit of NAD^+^ boosting compounds for improving cognitive function in ML/O adults is less clear, which has recently been reviewed in detail [101].

## Lifestyle and Select Pharmacological Strategies for Targeting the Shared Mechahisms of Peripheral Vascular Dysfunction and Brain Aging

**Figure 5:**
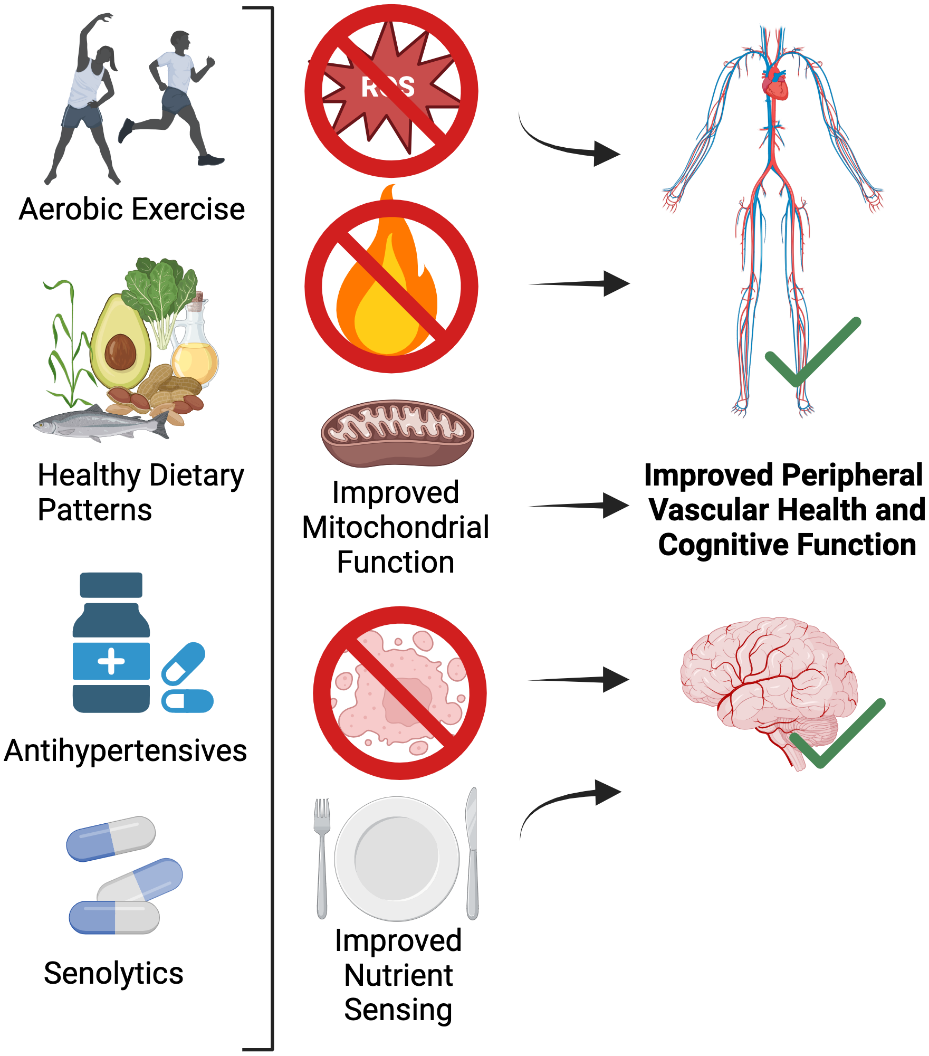
Lifestyle and select pharmacological strategies for targeting the shared mechanisms of peripheral vascular dysfunction and brain aging.

First-line therapy for improving peripheral vascular health with aging is increased physical activity and aerobic exercise [102]. However, select dietary and pharmacological (natural and synthetic) interventions have also emerged as putative therapies for improving age-related peripheral vascular function [35]. Growing evidence suggest that these interventions may also improve cognitive function and reduce the risk for dementia [103]. Below, we describe evidence (also shown in Table 1) supporting the role for increased physical activity and aerobic exercise, as well as certain whole dietary patterns and pharmacological agents for improving both peripheral vascular and cognitive function with aging (Figure 5).

**Table 1:** Clinical evidence regarding the potential efficacy of pharmacological and lifestyle interventions for improving peripheral vascular function and brain health with aging.

**Intervention**	**Population**	**Clinical outcomes**	**References**
**Lifestyle**			
*Aerobic Exercise*	Healthy midlife/older adults	- Improved endothelial function in men - Inconsistent improvements in endothelial function in estrogen deficient postmenopausal women - Reduced large elastic artery stiffness	Most recently reviewed in [109]
*High-Resistance Inspiratory Muscle Strength Training*		- Improved cerebrovascular endothelial function - Improved peripheral vascular endothelial function - No change in large elastic artery stiffness	[111, 149]
*Time-Restricted Feeding*	Healthy midlife/older adults (55-79 years; n=14)	- No influence on peripheral vascular function	[136]
*Intermittent Fasting*	Adults 35-75 years with obesity	- Enhanced hippocampal neurogenesis and memory	[138]
**Pharmacological**			
***Targeting Excessive Inflammation***			
*Salsalate*	Healthy midlife/older adults (50-79 years; n=9)	- Reduced aortic stiffness	[55]
*Salsalate*	Overweight or obese midlife/older adults (52-68 years; n=14)	- Improved peripheral vascular endothelial function	[56]
*Salsalate*	Healthy midlife/older adults (50-75 years; n=16)	- Improved peripheral vascular endothelial function	[150]
*Curcumin*	Healthy midlife/older adults (45-74 years; n=20)	- Improved peripheral vascular endothelial function - No influence on large elastic artery stiffness	[58]
***Targeting Excessive Mitochondrial Oxidative Stress***			
*MitoQ*	Healthy midlife/older adults (60-79 years; n=20)	- Improved peripheral vascular endothelial function - Reduced aortic stiffness in those with elevated aortic stiffness at baseline	[70]
***Targeting Reduced NAD^+^ Bioavailability***			
*Nicotinamide Riboside*	Healthy midlife/older adults (55-79 years; n=24)	- Treatment was safe - Reduced aortic stiffness - No influence on endothelial function	[93]
*Nicotinamide Riboside*	Adults with Parkinson’s disease (mean age 64 years; n=30)	- Treatment was safe - Modulated cerebral metabolic function	[151]
***Targeting Reduced Nitric Oxide Bioavailability***			
*Inorganic Nitrite*	Healthy midlife/older adults (50-79 years; ^a^n=49; ^b^n=9)	- ^a,b^Improved peripheral vascular endothelial function - ^b^Reduced large elastic artery stiffness	a - [142] b - [141, 142]

### Physical activity/aerobic exercise

Increased physical activity, by way of aerobic exercise, is considered first-line therapy for improving peripheral vascular function with aging [102], particularly in ML/O adult men, with supportive but mixed evidence regarding its efficacy for improving peripheral vascular function in estrogen-deficient postmenopausal women [104]. The beneficial effects of aerobic exercise on the peripheral vasculature have shown to be directly mediated by or associated with reduced inflammation [55, 105–107], improved mitochondrial function [66, 106, 108] and lower burden of cellular senescence [84], which has been reviewed in detail elsewhere [109].

The mechanisms by which physical activity/aerobic exercise improve aspects of brain health/cognitive function are less clear; however, evidence points towards the fact that increased physical activity [110] and other forms exercise training (e.g., high-resistance inspiratory muscle strength training) [111] promote increased cerebrovascular function in ML/O adults. These precise mechanisms are under investigation, but evidence shows that exercise increases circulating levels of the myokine Irisin, which is associated with improved cognitive function [112, 113]. In addition, physical activity increases circulating levels of brain-derived neurotropic factor (BDNF), which heightens neuroplasticity [114]. Therefore, it has been argued that physical activity/exercise may be the most effective way to improve both vascular health *and* cognitive function [103]. Additionally, recent evidence suggests that aerobic exercise might be an effective intervention to reduce dementia risk, although more work needs to be completed in this area [115].

### Dietary approaches

Nutrition can also have a profound impact on peripheral vascular health. Interestingly, most nutritional interventions/guidelines that improve peripheral vascular health also improve brain health. Evidence from human trials show that these improvements are mediated by numerous cellular and molecular pathways that intersect with the “hallmarks” of aging, including reduced inflammation, improved mitochondrial function, and lower burden of cellular senescence [3]. Below, we highlight select dietary approaches that have emerged as promising strategies for improving both peripheral vascular and cognitive function with aging.

#### Mediterranean diet

The Mediterranean diet, characterized by limited intake of red meat, saturated fats, and dairy products with high intake of fruits, vegetables, whole grains, beans, nuts/seeds, and olive oil (monounsaturated fats) [116], lowers the risk for CVD [117]. This dietary pattern is associated with reduced oxidative stress and inflammation (likely mediated by greater intake of soluble fiber and antioxidant-rich food sources) [118, 119] and reduced cellular senescence burden in the peripheral vasculature [120], thus potentially explaining its benefit on peripheral vascular health. Indeed, consumption of a Mediterranean-style diet has shown to directly lower aortic stiffness in ML/O adults [121]. Systematic reviews have also suggested that long-term adherence to this dietary pattern protects against cognitive decline with aging [122] and more recent evidence suggests that this diet is associated with less postmortem LOAD pathology (e.g., Aβ plaques) [123].

#### Japanese dietary patterns

Traditional Japanese diets are similar to the Mediterranean diet and contain high amounts of vegetables, fruits, legumes, soy, and omega-3 fatty acids, but low amounts of red meat, saturated fats, and dairy products [124, 125]. These diets are high in antioxidant-rich foods which may partly explain the lower CVD risk associated consumption of these diets [126–128]. Moreover, these diets are traditionally lower in sodium, which could directly augment peripheral vascular function in ML/O adults [129]. In addition to improving peripheral vascular health, recent prospective evidence shows that long-term adherence of this dietary pattern may be associated with decreased risk of dementia in older adults [130], although more carefully controlled trials are necessary to assess the links between this dietary pattern and dementia risk.

#### The Finnish geriatric intervention study to prevent cognitive impairment and disability [FINGER] diet

The FINGER nutritional intervention was a double-randomized controlled trial in which individuals with marked CVD and dementia risk factors were assigned to a two-year multidomain intervention consisting of controlled dietary guidelines, supervised exercise, cognitive training, and CVD risk monitoring. The diet consisted of limited protein intake (10–20% of daily energy), low fat intake (25–35% daily energy with <10% from saturated sources and >15-30% from unsaturated fat), and moderate carbohydrate intake (45–55% daily energy with <10% from refined sugar). This diet also calls for high fiber intake and limited salt/alcohol consumption [131]. In addition to improving well-established CVD risk factors (e.g., glucose and BMI), adherence to this diet also improved cognitive function in older adults as measured by a battery of tests [131]. However, it remains to be determined whether this diet directly improves peripheral vascular function, but it is likely, considering the abundance of antioxidant-rich foods and low sodium content.

#### Intermittent fasting

Intermittent fasting [IF] is a type of time restricted eating (extended time with little or no energy intake), which is thought to promote healthy aging through activation of nutrient sensing pathways (e.g., enhanced sirtuin activity and reduced signaling through the mammalian target of rapamycin pathway) [132, 133]. There are various types of IF including prolonged fasting, alternate day fasting, and time-restricted feeding [132, 134], but interestingly not all of these paradigms have been shown to improve peripheral vascular function and reduce CVD risk [135, 136]. These results may be partly explained the varying durations (e.g., 6 hour vs 24 hour fast) of the each intervention; however, robust evidence shows that IF can reduce oxidative stress and inflammation, improve mitochondrial function, and lower cellular senescence burden [137], which collectively could confer benefits in peripheral vascular function. Recent evidence also shows that IF benefits cognitive function, possibly via increased hippocampal neurogenesis in humans [138], and may also lower risk of dementia [139]. In this regard, more carefully controlled trials are needed to determine optimal length and duration of fasting paradigms.

## Pharmacological Agents

### NO-boosting compounds (e.g., Sodium nitrite; Nitrate-rich beet root juice)

As Nitric oxide (NO) is a major mediator of peripheral vascular function and its bioavailability decreases with advancing age, interventions aimed at directly enhancing NO bioavailability have gained much attention. A way to accomplish this is to target the nitrate-nitrite-nitric oxide pathway, which can be accomplished by directly supplementing with nitrite. For example, oral consumption of sodium nitrite has shown to directly restore peripheral vascular function in old mice [140] and ML/O adults [141, 142], by improving mitochondrial function [142]. Moreover, oral consumption of nitrate-rich beet root juice has shown to augment peripheral vascular function in postmenopausal women [143]. In addition to the improvements in peripheral vascular function, sodium nitrite supplementation has shown promise for improving cognitive function in ML/O adults [144].

### Antihypertensives

As described above, the links between blood pressure and dementia risk are surprisingly unclear [145]. Several uncertainties surround current guidelines for the management of blood pressure to maintain brain health with aging, including optimal blood pressure in mid-life (including blood pressure variability) and the types of antihypertensive medications [146]. Nonetheless, evidence suggests that proper management of blood pressure in mid-life is critical in delaying cognitive decline [147] and recent evidence suggests that antihypertensive medications may even protect against dementia [148]. Clinical/neuropathological data also suggest that the use of antihypertensives reduce cerebrovascular disease and Aβ pathology [152]. The mechanisms linking hypertension to dementia risk are unclear, but most likely involve aortic stiffening at the level of the peripheral vasculature and white matter lesions and cerebromicrovascular injury at the level of the brain [153], which can contribute to cortical atrophy [154].

### Senolytics

As noted above, increased cellular senescence burden contributes to both peripheral vascular and cognitive dysfunction with aging, and synthetic pharmacological senolytics have shown promise (mostly in preclinical models) for enhancing function. However, there is limited translational potential of these senolytic approaches (e.g., ABT-263 [Navitoclax] and Dasatanib + quercetin [D+Q]) due to potentially adverse safety profiles in healthy ML/O adults; however, these senolytic interventions may we warranted in patient populations with severe disease conditions (e.g., idiopathic pulmonary fibrosis) [155]. To overcome this barrier, natural food-derived senolytic compounds have emerged as a promising strategy, and of the compounds screened to date, fisetin (commonly found in foods such apples, strawberries and onions) has shown the greatest promise [156]. Indeed, we recently found that oral intermittent (one week on; two weeks off; one week on) administration of fisetin improved carotid artery endothelial function and lowered aortic stiffness in old mice [157], and these results are currently being translated to ML/O adults to determine if oral intermittent fisetin supplementation can improve peripheral vascular endothelial function (NCT06133634).

In an ongoing clinical trial assessing the safety and efficacy of D+Q-based senolytic therapy for modulating the progression of Alzheimer’s Disease in patients with a mild Alzheimer’s Disease diagnosis (NCT04063124), it is clear that the senolytic compounds enter the cerebral circulation (e.g., the cerebrospinal fluid) but the study is ongoing, thus it remains to be determined if senolytic therapy with D+Q, can improve Alzheimer’s Disease-related phenotypes [158]. Importantly, D+Q appeared to be safe in the preliminary analysis of this study, which could shift the way the field views the translational potential of D+Q (i.e., beyond only dosing patients with severe disease states). Currently, no studies are ongoing or have been completed assessing the efficacy of oral fisetin supplementation for improving cognitive function.

## Conclusions, Research Gaps and Future Directions

**Figure 6:**
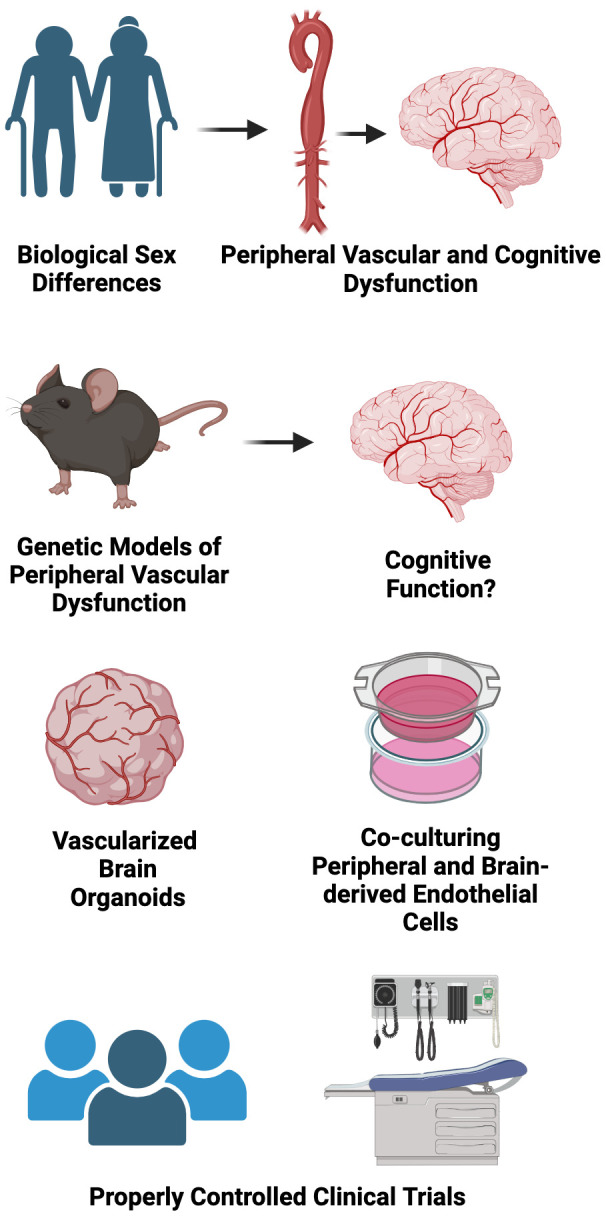
Conclusions, research gaps, and suggested future directions.

Aging is the major risk factor for CVD and Alzheimer’s disease/related dementias. Increased CVD risk with aging is due importantly to the development of peripheral vascular dysfunction, characterized by large elastic artery stiffening and endothelial dysfunction. Currently, the pathophysiological sequalae of brain aging is not completely understood, but changes in peripheral vascular function could mediate the progression of Alzheimer’s disease and related dementias. In this review, we discussed the shared cellular/molecular aging “hallmarks” underlying both peripheral vascular function and cognitive impairment, and how these “hallmarks” may be viewed as viable therapeutic targets for reducing CVD and Alzheimer’s disease and related dementias risk in ML/O adults. Finally, we highlight established lifestyle and select pharmacological interventions that could be used to target these “hallmarks” to improve peripheral vascular and cognitive function with aging. There remain several important knowledge gaps in the field; the following represent some potential future biomedically significant directions for research related to healthy CV and brain aging (Figure 6).

### Considering sex as a biological variable

Biological sex is a non-modifiable risk factor for peripheral vascular dysfunction and various types of dementia. For example, ML/O adult estrogen deficient postmenopausal women are at greater risk of developing CVD [159] and vascular dementia and LOAD [160, 161] relative to age-matched men. The precise mechanisms underpinnings these phenotypes are not completely clear but may in part be explained the progressive loss of peripheral vascular function over the menopausal transition [162, 163], as a similar loss in gonadal function does not consistently occur in age-matched men. Moreover, the greater prevalence of dementia in women could be explained by the fact that women survive to older ages when compared to men [164], or due to the potential protective effect of estrogen on brain function, as physiological levels of estrogen mitigate excessive oxidative stress [165, 166]. Thus, there is a clear need to more comprehensively understand how biological sex and gonadal status influence the relation between peripheral vascular function and cognitive health with aging and the cellular/molecular mechanisms underpinning these differences.

### Basic science experimental approaches to look at the crosstalk between the peripheral vasculature and the brain through the lens of aging “hallmarks”

In recent years, there has been accelerated development and utility of organoid models to better understand mechanisms underlying *in vivo* physiology. For example, vascularized brain organoids have emerged as an experimental tool for providing novel insight into human brain development and disease [167], such that these organoid models can be manipulated *ex vivo* and successfully grafted *in vivo*. Moreover, sophisticated experimental approaches using co-culture systems (i.e., co-culturing peripheral vascular cells with brain cells) may be used to directly interrogate how adverse changes in aging “hallmarks” in peripheral vascular cells influence the health and function of brain cells.

### Genetic models to better understand the role of peripheral vascular dysfunction in mediating brain aging

Several pre-clinical genetic models have been utilized to better understand how peripheral vascular dysfunction mediates brain aging [168]. One such example is the elastin haploinsufficient (single deletion of the *Elastin* gene) mouse model. Phenotypically, this model exhibits accelerated large elastic artery (e.g., aorta) stiffening, which allows for the opportunity to study the influence of aortic stiffening independent of advancing/advanced age [169]. Interestingly, results from studies using this model indicate that large elastic artery stiffness has a greater effect on inducing endothelial dysfunction in cerebral arteries when compared to peripheral arteries and that these effects are likely mediated by reduced NO bioavailability and excessive ROS-related oxidative stress [169]. Additional studies using this mouse model show that large-artery stiffness impairs spatial memory [170]. In addition to the elastin haploinsufficient mouse, the Fibrollin-1 haploinsufficient (single deletion of the *Fibrollin-1* gene, which provides structural support to elastic tissue) mouse model has provided some insight into how large elastic artery stiffness affects the brain [171]. Interestingly, this model exhibits higher than average levels of reactive oxygen species in brain vasculature, but it is unclear if this effect is due to increased large artery stiffness or as a direct result of the genetic deletion [42]. Nonetheless, the continued use of these existing mouse models, or the development of new models (perhaps a model with elastin haploinsufficiency strictly in the peripheral vasculature), would provide a key opportunity to more comprehensively understand the contributions of peripheral vascular dysfunction to brain aging.

### Properly powered placebo-controlled randomized clinical trials to determine the efficacy of aging “hallmark”-targeted therapies for improving peripheral vascular function and brain health with aging

Although there are numerous ongoing clinical trials seeking to determine the efficacy of targeting the “hallmarks” of aging to improve peripheral vascular and cognitive function, many of these studies strictly focus on one outcome or the other. Given the influence of peripheral vascular function on brain health discussed throughout this review, it would be highly advantageous to study both concomitantly. There are obvious mechanistic limitations to this approach, as one could not clearly discern whether the intervention-mediated improvements in brain health were directly driven by improvements in peripheral vascular function or via a direct effect on the brain; however, these studies could provide essential information regarding how the aging “hallmarks” integrate into the temporal progression of peripheral vascular function-induced changes in brain health.
